# Advertisements of ultra-processed products outside food outlets: field evidence from Montevideo, Uruguay

**DOI:** 10.1017/S1368980025000254

**Published:** 2025-03-19

**Authors:** Gastón Ares, Florencia Alcaire, Gerónimo Brunet, Tobias Otterbring, María Costa, Sofía Verdier, María Rosa Curutchet, Luciana Bonilla, Sergio Turra, Fernanda Risso, Leticia Vidal

**Affiliations:** 1 Sensometrics & Consumer Science, Instituto Polo Tecnológico de Pando, Facultad de Química, Universidad de la República, By Pass de Rutas 8 y 101 s/n. CP 91000, Pando, Uruguay; 2 Espacio Interdisciplinario, Universidad de la República, José Enrique Rodó 1843, CP 11200, Montevideo, Uruguay; 3 School of Business and Law, Department of Management, University of Agder, Universitetsveien 17, Kristiansand, Norway; 4 Instituto Nacional de Alimentación, Ministerio de Desarrollo Social, Piedras 165, CP 11000, Montevideo, Uruguay; 5 Escuela de Nutrición, Universidad de la República, Av. Ricaldoni S/N, CP 11600, Montevideo, Uruguay

**Keywords:** Food environment, Food marketing, Out-of-home marketing, Outdoor marketing, Commercial determinants of health

## Abstract

**Objectives:**

To evaluate the prevalence of advertisements for ultra-processed products outside food outlets in Montevideo (Uruguay) and explore the patterns of these advertisements across areas with different socio-economic statuses (SES).

**Design:**

Cross-sectional field survey of advertisements of ultra-processed products outside food outlets. The percentage of outlets featuring any type of advertisement of ultra-processed products on the exterior part of the outlets was calculated, at the aggregate level and separately by type of outlet and type of product. Comparisons were made considering the SES of the tract where outlets were located.

**Setting:**

106 census tracts in Montevideo, differing in geographical location and SES.

**Participants:**

Outlets selling foods and beverages, located within the selected census tracts.

**Results:**

30·7 % of the 1217 food outlets identified in the field survey featured some type of exterior advertisement of ultra-processed products. Sweetened beverages (specifically soda) were the most frequently advertised ultra-processed product category, followed by ice cream. After adjusting for the type of outlet, medium SES tracts exhibited the highest prevalence of ultra-processed product advertisements outside food outlets (36·0 %). Differences in the prevalence of advertisements of specific categories with SES were also found, which may reflect variations in the types and characteristics of food outlets.

**Conclusions:**

Results from this work suggest the frequent presence of exterior advertisements of ultra-processed products and highlight the need to develop effective policies to reduce their use as part of a comprehensive set of strategies to discourage the consumption of ultra-processed products.

Modern food systems are oriented towards the production of ultra-processed products^(1,2)^, i.e. ‘formulations of ingredients, mostly of exclusive industrial use’^(3)^. Their consumption has been associated with higher risk of several adverse health outcomes, including obesity, hypertension, cardiovascular diseases, type 2 diabetes, cancer, depression and all-cause mortality^(4)^. Sales of these products have increased in recent years, particularly in emerging countries^(5)^, due to several factors, including the commercial practices developed by the food industry to increase demand and market coverage^(6)^.

Food marketing is one of those commercial practices, which makes products more salient in consumers’ mind, influences product-related attitudes and shapes purchasing behaviour^(7,8)^. According to the American Marketing Association, such commercial practices include a wide range of activities for creating, communicating, delivering and exchanging offerings^(9)^. Out-of-home marketing is one of the oldest and most widespread forms of marketing communications^(10)^. It refers to ‘any advertising media found outside of the home but typically not inside a store’^(11)^, including advertising on public buildings, public facilities and structures, as well as advertising displayed outside outlets^(10)^. This type of marketing can be used to target selected consumer segments at a specific time and place where they are more likely to pay attention, often acting as a reminder of which particular products or brands to purchase^(12)^. Although research on out-of-home marketing is still limited, it has been reported to be frequently used to promote unhealthy foods^(13)^. Two studies have reported associations between exposure to out-of-home marketing and a higher likelihood of consuming products marketed through this out-of-home medium, such as soda and confectionery^(14,15)^.

In particular, the placement of advertisements outside of food outlets has the potential to target potential shoppers, creating exposure and influencing purchase decisions in places where products are sold^(12)^. This is particularly true for hedonic products that give the buyer some sort of sensory pleasure, as products associated with positive affective states are vastly over-represented among those that consumers purchase, prefer or choose on impulse^(16–18)^. Notwithstanding such findings, few studies have analysed the prevalence of advertisements outside food outlets^(13)^. Isgor *et al.*, through in-store audits conducted on a nationwide sample of food outlets in the USA, found that 58·6 % of supermarkets and grocery stores, as well as 73·0 % of limited-service stores, displayed exterior advertisements for foods and beverages^(19)^. Barnes *et al.*, through in-store audits on a random sample of licensing lists, reported that 46 % of the small and nontraditional food stores in Minneapolis-St-Paul included at least one exterior advertisement of unhealthy foods^(20)^. Importantly, as far as can be ascertained, no study has reported the prevalence of exterior food advertisements in emerging countries from the majority world, i.e. societies in Asia, Africa, Latin America and the Caribbean where most humans live^(21)^. Such knowledge gaps are important to address from a generalisability perspective, as findings from developed countries might differ dramatically from those obtained in other parts of the world^(22)^.

Research conducted predominantly in developed countries in North America, Europe and Oceania has shown that low-income citizens and other vulnerable population groups tend to be disproportionately exposed to the marketing of unhealthy foods, which may worsen existing inequities in diet quality and health^(23)^. Indeed, previous studies have shown that outdoor marketing of unhealthy foods is more prevalent in low-income communities^(24–28)^. Yet, the evidence on socio-economic differences in the prevalence of advertisements outside stores is limited to only one study conducted in the USA and hence a developed country^(19)^. Isgor *et al.* found that food and beverage advertisements were more prevalent in supermarkets and grocery stores located in low-income areas compared with high-income areas (51·6 % *v*. 34·3 %). This trend extended to one specific unhealthy food category: the prevalence of advertisements specifically for regular soda was significantly higher in low-income areas (25·1 % *v*. 10·4 %)^(19)^.

In this context, the aims of this work were to (i) evaluate the prevalence of advertisements of ultra-processed products outside food outlets in Montevideo, the capital city of Uruguay, an emerging Latin American country pertaining to the majority world and (ii) explore the patterns of these advertisements across areas with different socio-economic status (SES). Results are expected to contribute to a more in-depth understanding of the marketing practices implemented by the food industry to influence consumer behaviour and encourage consumption of ultra-processed products.

## Methods

This study was part of a larger project on the retail food environment, approved by the ethics committee of the School of Chemistry (Universidad de la República, Uruguay). It relied on a cross-sectional field survey of outlets selling foods and beverages in Montevideo, the capital city of Uruguay. Uruguay is a high-income country located in the south-eastern region of South America, between Argentina and Brazil. Montevideo is the most populated city with 1 670 545 inhabitants and has an area of approximately 526 km^2^. While Uruguay shares some similarities with developed countries, particularly in terms of democratic governance, and urbanisation, it differs in key areas such as industrialisation, economic development and education. The country has a strong agricultural export sector but is less industrialised than developed countries, which have a greater focus on advanced technology and diversified industrial economies. Regarding economic development, although Uruguay is categorised as a high-income country, its gross domestic product (23 090 US dollars per capita) is markedly lower compared with developed countries^(29)^. In Uruguay, the average learning-adjusted years of schooling fall 2–3 years behind those of developed countries in North America, Europe and Oceania^(30)^.

Local regulations require all forms of outdoor marketing to be approved by the city government^(31)^. However, at the time of data collection, there were no national or local restrictions specifically targeting outdoor food marketing. The only existing food marketing regulation was a prohibition on marketing foods high in sugar, total fat, saturated fat or Na within school settings^(32)^.

### Sampling

For statistical purposes, the city of Montevideo is divided into census tracts, which are geographic units used for analysing data from official surveys and censuses. In urbanised areas, census tracts typically consist of a group of city blocks, while in non-urbanised areas, they represent portions of land with clear physical boundaries and small, dispersed population groups.

**Figure 1.**
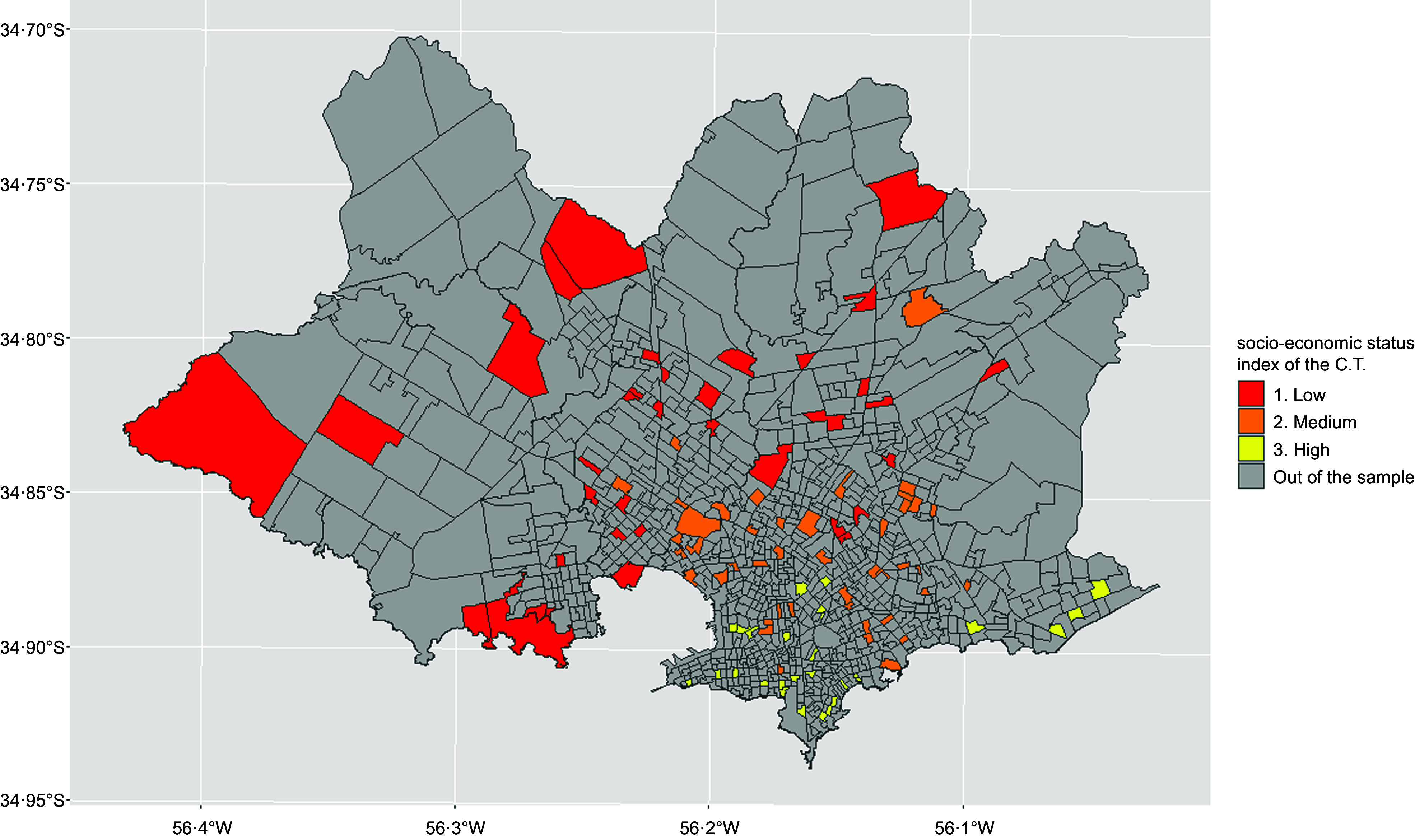
Distribution of the census tracts included in the sample (*n* 106), according to their socio-economic status (SES), in the city of Montevideo (Uruguay).

Census tracts were used to select the area of analysis. A sample of 106 census tracts was obtained using probability proportional-to-size sampling. The eight city’s municipalities were considered as strata. The size of the sample accounted for 10 % of all the census tracts and covered an area of 62·43 km^2^. The census tracts were distributed along the city and differed widely in their SES index (Figure 1). The socio-economic index of each census tract corresponded to the score granted to the neighbourhood where the census tract was located in the standard methodology used in the country for the estimation of household SES^(33)^. The score ranges between 0 and 13. Using these scores, three groups of census tracts were identified: low SES (*n* 36, indexes ranging from 0 to 4), medium SES (*n* 44, indexes ranging from 5 to 9) and high SES (*n* 26, indexes ranging from 10 to 13).

### Data collection

A field survey was conducted to identify all the food outlets located within each of the 106 census tracts. The field survey aimed to achieve three key objectives: (i) validate secondary databases of the retail food environment, (ii) characterise food outlets and (iii) estimate the prevalence of advertisements of ultra-processed products outside food outlets. While this study focuses on the third objective, the data collection procedures are described comprehensively to provide full context.

Teams of two observers walked all the streets within the census tracts. They were given a list of all the food outlets listed on administrative records from the national and local governments and Google maps within each of the census tracts^(34)^. Observers were asked to walk all the streets within the census tracts and register all the outlets located on both sides of the streets. For the boundaries of the tract, observers were asked to only register the outlets located on the side of the street included in the tract. For outlets listed in the database, they had to check the available information and register the existence of marketing of ultra-processed products outside the outlets. For outlets not listed in the database, they had to register the outlet’s name, address, type of outlet and the existence of exterior marketing of ultra-processed products. The following types of outlets were considered in the data collection form: supermarkets; grocery stores; restaurants, bars or takeaways; kiosks; bakeries; fruit and vegetable stores; butchers’, poultry shops or fishmongers’; other specialised stores (e.g. cheese store, delicatessen); pharmacies; street vendors; street markets and non-storefront outlets (i.e. outlets not primarily focused on selling foods, such as gyms or laundromats).

Each pair of observers had to register whether each outlet had any type of exterior advertisement of ultra-processed products. This included billboards, posters, brand logos or any type of signage located on the exterior of the building, its premises or the sidewalk in front of the outlet. A Yes/No question was used for this purpose. When exterior advertisements of ultra-processed products existed, they had to complete an open-ended question to describe the advertised brand and product. In case of doubts regarding whether the advertised products were ultra-processed, they were instructed to include the information. Data were registered using Compusense Cloud (Compusense Inc., Guelph, Canada).

Observers were trained on how to use the data collection instrument and the maps of the census tracts for data collection. The training included the definition of ultra-processed products developed by Monteiro *et al.* ^(3)^, as well as a list of categories frequently regarded as ultra-processed^(35)^ (see online supplementary material, Supplemental Table 1). The instrument was pilot-tested in two census tracts, and no substantial changes were made subsequently. Only minor modifications to the form were introduced to facilitate data collection. The field survey was completed on weekdays in the morning (09.00–13.00) or the afternoon (15.00–17.00), between September 2023 and February 2024. For farmers’ markets, a part of the fieldwork was conducted on weekends.

After completing data collection, a quality check was conducted in three randomly selected census tracts. A different group of observers repeated the data collection, and no discrepancies were identified between the two datasets. Consequently, the data quality was considered satisfactory.

### Data analysis

A word cloud was used to obtain a graphical representation of the advertisements captured by the observers, without any type of grouping or categorisation. Responses were translated from Spanish to English, except for brand names. Typographical errors were corrected and stop words or terms indicating the location of the advertisements were excluded.

Content analysis based on inductive coding was used to categorise the advertised ultra-processed products identified in the field survey^(36)^. One of the researchers, with a background in food science and technology and extensive experience in content analysis, developed a coding frame to group the advertisements into categories. The coding process involved verifying whether the products listed by observers met the criteria to be regarded as ultra-processed, based on the definition by Monteiro *et al.* ^(3)^ Of the 561 entries, six were excluded because they referred to alcoholic beverages (wine or beer), which were not classified as ultra-processed products. Brand advertisements were categorised based on the types of products sold under the brand name. No advertisements were found to advertise brands commercializing products spanning multiple categories within the coding frame. Once the coding was finalised, it was revised by a second researcher and no changes were made. Binary variables were created to indicate whether each food outlet featured any type of exterior advertisement for each of the product categories.

The percentage of outlets displaying exterior advertisements of ultra-processed products was calculated at the level of all census tracts and separately for tracts with different SES (low, medium, high). Results were also disaggregated by type of outlet and advertised product category.

Logistic regressions were used to compare the prevalence of exterior advertisements of ultra-processed products in census tracts with different SES levels. A binary variable indicating the existence of exterior advertisements was considered a dependent variable, whereas the SES of the tract where the outlet was located (low, medium, high) was considered as an explanatory factor. Analysis of deviance of each model was performed using chi-square tests considering a significance level of 0·05. Tukey’s test was used for post hoc comparisons. Regressions were run at the aggregate level and separately for each type of outlet and advertised product.

The distribution of outlets with exterior advertisements was analysed based on outlet type across census tracts of different SES levels (low, medium and high). Only types of outlets with more than five outlets for the three SES groups were considered in the analysis. A chi-square test was used to compare the distributions. All data analyses were performed using R software^(37)^ version 4.2.0.

## Results

A total of 1217 food outlets were identified across the 106 census tracts: 453 in low SES tracts, 405 in medium SES tracts and 359 in high SES tracts. As shown in Table 1, supermarkets or grocery stores and restaurants, bars or takeaways were the most prevalent types of outlets, followed by kiosks and bakeries. Most of the food outlets (91·9 %) sold ultra-processed products. The sale of ultra-processed products was frequent in all types of outlets, including fruit and vegetable stores and street markets. The sole exception was street vendors, among which the prevalence of selling ultra-processed products was notably lower (35·5 %).

**Table 1.** Number of different types of food outlets in Montevideo (Uruguay) and percentage of outlets selling ultra-processed products (in parenthesis), at the aggregate level and by socio-economic status (SES) of census tracts

Type of outlets	All census tracts (*n* 106)	Low SES tracts (*n* 36)	Medium SES tracts (*n* 44)	High SES tracts (*n* 26)
Supermarkets or grocery stores	364 (97·8)	189 (97·9)	109 (96·3)	66 (100·0)
Restaurants, bars or takeaways	301 (98·3)	52 (98·1)	97 (97·9)	152 (98·7)
Kiosks	147 (95·9)	63 (98·4)	56 (94·6)	28 (92·9)
Bakeries	109 (77·1)	25 (80·0)	46 (82·6)	38 (68·4)
Fruit and vegetable stores	74 (86·0)	40 (97·7)	23 (93·9)	11 (41·2)
Butchers’, poultry shops or fishmongers’	40 (92·5)	24 (95·8)	9 (100·0)	7 (71·4)
Other specialised stores	53 (76·9)	22 (85·7)	11 (83·3)	20 (81·3)
Pharmacies	46 (87·0)	10 (100·0)	16 (100·0)	20 (70·0)
Non-storefront outlets	32 (84·8)	10 (100·0)	14 (86·7)	8 (62·5)
Street vendors	31 (35·5)	15 (40·0)	13 (38·5)	3 (0·0)
Street markets	20 (90·0)	3 (66·7)	11 (90·9)	6 (100·0)
Total	1217 (91·9)	453 (94·4)	405 (92·6)	359 (88·1)

For each type of outlet, the percentage of those selling ultra-processed products is indicated in parentheses.

**Figure 2.**
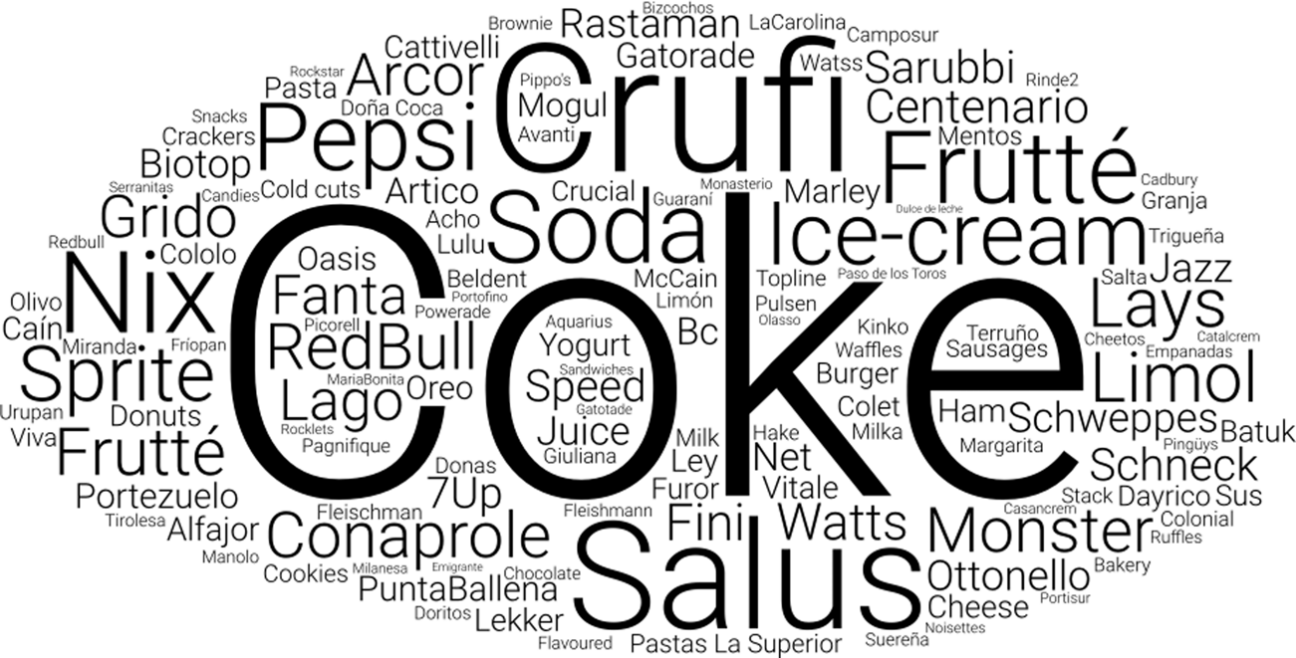
Word cloud showing the advertisements of ultra-processed products and brands registered by observers in the exterior of food outlets in the city of Montevideo (Uruguay).

Figure 2 presents the responses recorded by observers during the field survey, documenting exterior advertisements of ultra-processed products at food outlets. The most frequently mentioned words corresponded to brands of sweetened beverages, ice creams and other dairy products. Brand names corresponded to global, regional and national brands. The brand ‘Coke’ was the most frequently identified, appearing in advertisements at 148 food outlets (12·2 %), followed by ‘Crufi’, a popular Uruguayan ice cream brand owned by Froneri International (*n* 86, 7·1 %) and ‘Salus’ (*n* 48, 3·9 %), a Uruguayan brand owned by Danone Ambev, which markets flavoured water and soda (excluding mineral water, which was not part of the survey).

Considering all the census tracts, 30·7 % of the food outlets featured some type of exterior advertisement of ultra-processed products. Supermarkets or grocery stores and kiosks were the outlets with the largest prevalence of exterior advertisements, followed by specialised stores and bakeries (Table 2). Conversely, exterior advertisements of ultra-processed products were not frequent in fruit and vegetable stores, street vendors or street markets and pharmacies.

**Table 2.** Percentage of food outlets featuring some type of exterior advertisement of ultra-processed products in the city of Montevideo (Uruguay), for the 106 census tracts included in the sample and separately for tracts of different socio-economic status (SES)

	All census tracts (*n* 106)	Low SES tracts (*n* 36)	Medium SES tracts (*n* 44)	High SES tracts (*n* 26)	*P*-value
All outlets and food types	30·7	32·1a	36·0a	23·1b	< 0·001
Type of outlet					
Supermarkets or grocery stores	53·3	49·2	61·5	51·5	0·118
Kiosks	44·2	31·7b	50·0a,b	60·7a	0·195
Other specialised stores	35·8	13·6b	45·5a,b	55·0a	0·138
Bakeries	27·5	24·0	32·6	23·7	0·604
Restaurants, bars or takeaways	16·3	30·8a	22·7a	7·2b	< 0·001
Butchers’, poultry shops or fishmongers’	15·0	12·5	33·3	0	0·164
Non-storefront outlets	12·0	20·0	13·3	0	0·451
Street markets	10·0	0	9·1	16·7	0·759
Street vendors	6·5	6·7	7·7	0	0·896
Fruit and vegetable stores	4·1	2·5	8·7	0	0·38
Pharmacies	2·2	0	6·3	0	0·401
Advertised product category					
Sweetened beverages	19·7	16·2b	25·6a	17·2b	< 0·001
Ice cream	10·0	15·0a	9·1b	4·7c	< 0·001
Cold cuts	3·1	4·2a	3·7a,b	1·1b	0·030
Bakery products (e.g. cookies, crackers and alfajor*)	2·5	3·1	2·2	2·2	0·646
Yoghurt or flavoured milk	1·0	0·6	1·2	1·1	0·673
Savoury snacks	0·9	0·7	1·2	0·8	0·669
Candies or chocolate	0·9	0·2	1·5	1·1	0·133
Frozen meals and products	0·6	0·2	0·7	0·8	0·449
Others (e.g. pie crusts, pasta)	1·1	2·0a,b	2·5a	0·8b	0·040

Percentages within a column with different letters are significantly different according to Tukey’s post hoc comparison test for a significance level of 0·05. *Alfajor is a traditional confectionary product, consisting of two layers of cookies separated by a layer of sweetened condensed milk or chocolate and covered with meringue or chocolate.

Sweetened beverages were the most frequently advertised ultra-processed product category (19·7 %), followed by ice cream (10·0 %). In particular, soda was the most frequently advertised product; 16·4 % of the food outlets featured exterior marketing of this product. The rest of the beverages were only advertised in less than 4 % of the outlets: flavoured water (3·4 %), energy drinks (2·6 %), isotonic beverages (0·5 %), juices and nectars (0·8 %) and powdered drinks (0·3 %). Other advertised products on the exterior of food outlets included cold cuts, bakery products, yoghurt or flavoured milk, savoury snacks, candy or chocolate and frozen meals and products.

At the aggregate level, the prevalence of advertisements of ultra-processed products outside food outlets differed as a function of the SES of the census tracts (Table 2). Outlets located in low and medium SES tracts were 1·57 [1·15—2·17] and 1·88 [1·37–2·59] times more likely to feature exterior advertisements of ultra-processed products compared with outlets located in high SES tracts, respectively. After adjusting for the type of outlet, medium SES tracts exhibited the highest prevalence of ultra-processed product advertisements outside food outlets. Outlets in low SES tracts were 41 % less likely to display such advertisements (OR = 0·60 [0·43–0·83]) compared with those in medium SES tracts. Similarly, outlets in high SES tracts were 42 % less likely (OR = 0·58 [0·41–0·83]) to feature these advertisements than those located in medium SES.

Significant differences in the prevalence of advertisements of ultra-processed products were also identified for specific types of outlets. As shown in Table 2, the prevalence of advertisements of ultra-processed products outside the outlets was lower in high SES tracts compared with medium and low SES for restaurants, bars and takeaways. Conversely, exterior advertisements of ultra-processed products tended to increase with SES for kiosks and specialised stores. The proportion of these outlets featuring exterior advertisements was significantly higher in high SES tracts compared with low SES tracts. However, no significant differences were observed between medium SES tracts and the other two SES categories.

**Figure 3.**
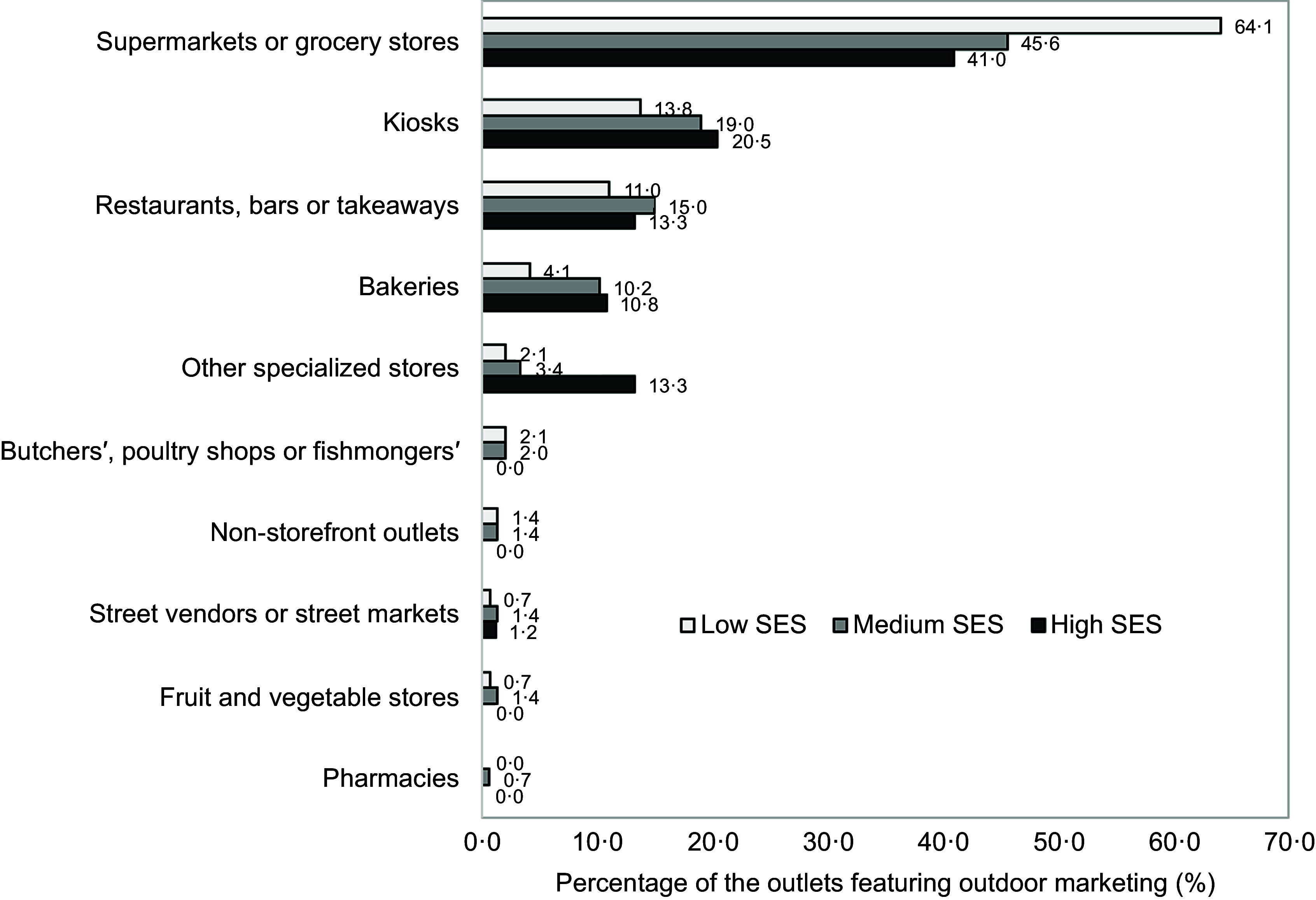
Distribution of outlets featuring outdoor advertisements of ultra-processed products within each group of census tracts (low, medium and high socio-economic status (SES)) in the city of Montevideo.

A significant association between the percentage of outlets featuring exterior advertisements of ultra-processed products and the SES of the tract was found (c^2^ = 28·1, p < 0·001). This finding indicates that the types of outlets contributing to such advertisements varied depending on the SES of the census tract. Figure 3 illustrates the varying contributions of different outlet types to exterior advertisements of ultra-processed products across SES tracts. In low-SES areas, supermarkets and grocery stores were the primary contributors, representing 64·1 % of outlets with exterior advertisements of ultra-processed products. In contrast, their contribution was lower in medium SES (45·6 %) and high SES tracts (41·0 %). Kiosks and bakeries played a larger role in medium and high SES areas, accounting for a larger share of the outlets featuring ultra-processed product advertisements. Additionally, specialised stores had their highest contribution in high-SES tracts, where they represented 13·3 % of outlets featuring these advertisements.

The prevalence of exterior advertisements for specific categories of ultra-processed products outside the outlets also significantly differed across SES. Marketing of sweetened beverages was most prevalent in medium SES tracts, whereas the prevalence of marketing of ice creams and cold cuts significantly decreased with SES (Table 2).

## Discussion

People are constantly exposed to a variety of marketing stimuli, including food packaging, out-of-home marketing, TV and other mass media and digital platforms, which are expected to influence their attitudes and behaviours^(13,38–42)^. An in-depth understanding of the marketing practices used by the food industry to promote ultra-processed products is needed to develop effective public policies to discourage their consumption. The present research focused on advertisements outside food outlets, one of the forms of out-of-home marketing that has the potential to influence decisions at the point of purchase.

Results showed that 30·7 % of all the food outlets identified in the field survey had exterior advertisements of ultra-processed products. Supermarkets, grocery stores and kiosks were the types of outlets most likely to feature these advertisements, with approximately half and two-fifths of the stores, respectively, including exterior advertisements of ultra-processed products. Considering that supermarkets and grocery stores are the outlets where citizens are expected to make most of their food purchases, results from this work suggest frequent exposure to exterior advertisements of ultra-processed products at the point of purchase. These advertisements may make specific products more salient in consumers’ minds when making their decisions, encouraging impulsive purchases^(8)^.

Direct comparison with other studies is not possible due to methodological differences related to the types of outlets and advertisements included. However, the prevalence of exterior advertisements of ultra-processed products in close connection to supermarkets, grocery stores and kiosks is similar to the value reported by Barnes *et al.* when analysing the prevalence of advertisements of unhealthy foods and beverages in small and non-traditional stores in Minneapolis, St. Paul (46 %)^(20)^.

The most frequently advertised brands were associated with transnational food manufacturing corporations or national brands owned by these corporations. These companies are known to employ diverse market strategies to expand and consolidate their influence, including acquiring local food manufacturing firms in foreign markets and making significant investments in marketing initiatives^(43,44)^. Sweetened beverages and ice cream were the categories most frequently advertised on the exterior parts of the examined outlets. These products typically contain excessive content of sugar and/or saturated fat as well as several food additives. In particular, sweetened beverages have been extensively associated with negative health outcomes^(45,46)^. A possible explanation for the frequent marketing of these product categories may be the trade promotion practices used by companies commercializing these products to shape marketing at outlets^(47)^, as fridges and freezers may be given to retailers in exchange for placing advertisements outside the outlets. Research on the factors underlying retailers’ decision to place advertisements outside the outlets may contribute to the development of public policies to reduce their prevalence.

Results from the present research showed differences in the prevalence and pattern of advertisements of ultra-processed products outside food outlets with the SES of the areas where they were located. At the aggregate level, food outlets in medium SES census tracts were more likely to feature exterior advertisements of ultra-processed products compared with those in low or high-SES areas. This result suggests that residents of medium SES neighbourhoods may face greater exposure to such advertisements at the point of purchase. While differences in advertisement prevalence across SES areas varied by outlet type, the data did not indicate a higher prevalence of exterior advertisements in low SES areas. These results contrast with previous studies from developed countries in North America, Europe and Oceania, which reported larger exposure to unhealthy food marketing among the most vulnerable populations^(19,24–28)^.

Differences in the prevalence of advertisements of specific products were also found across SES areas: advertisements of sweetened beverages outside outlets were most frequent in medium SES tracts, whereas advertisements of ice cream and cold cuts tended to reduce with SES. These findings may reflect variations in the types and characteristics of food outlets across different SES areas. Further research is required to understand the factors influencing the use of ultra-processed product advertisements in the exteriors of food outlets, especially in emerging countries. A deeper understanding of the practices employed by ultra-processed product companies to promote the use of exterior advertisements can provide valuable insights for shaping effective regulatory actions. Research is also needed to gain a deeper understanding of how SES influences exposure to different types of advertisements for unhealthy foods. This is particularly relevant considering that populations with low income and education levels have been reported to be more vulnerable to the persuasive effects of food marketing^(48,49)^.

### Strengths and limitations

The main strength of this study is its novelty, as it is one of the few to analyse advertisements outside of food outlets. Further, this study is the first conducted in the context of an emerging country in the majority world, the latter of which is the world region where most humans live, despite being rarely represented in academic research. As such, the findings reported herein build on and extend previous studies that have solely been conducted in developed countries, typically in English-speaking Western world regions, such as the USA, the UK, Canada, Australia and New Zeeland, with these countries only accounting for slightly more than 5 % of the world’s population^(50)^. Considering that the current results largely resemble those by Barnes *et al.* ^(20)^, this study contributes to offering considerable cross-cultural generalisability of prior findings that have been restricted to English-speaking developed countries.

From a methodological point of view, data were also collected through a field survey that included all the food outlets within 10 % of all the city’s census tracts. This allowed a comprehensive evaluation of the prevalence of exterior advertisements of ultra-processed products, thus contributing to more representative results and enhanced ecological validity^(51)^.

The study also has several limitations. During data collection, observers determined whether advertisements corresponded to ultra-processed products, but no photographs of the advertisements were taken. Although two different methods were employed to verify the reliability of the data, details about specific advertisements may have been overlooked. In addition, the content of the advertisements was not analysed, hindering a more fine-grained analysis of the power of exterior food marketing of ultra-processed products. In addition, the study was limited to advertisements outside stores, meaning that marketing inside stores and all other forms of out-of-home marketing were not considered. Further research is needed to obtain a more comprehensive analysis of the prevalence and characteristics of out-of-home marketing of ultra-processed products. Third, the sample size used in the present research is not enough to produce precise and accurate estimates of the prevalence of outdoor advertisements in food outlets, hindering the possibilities of generalising the results to the total area of the city. Finally, it is important to note that the study was conducted in a single city over a limited time frame, which constrains the generalisability of the findings. Additionally, the results may have been influenced by the seasonality of data collection, as it was conducted during spring and summer.

### Conclusions

Results from the present research indicate that advertisements of ultra-processed outside stores, particularly supermarkets, grocery stores and kiosks, are frequent in Montevideo (Uruguay). Considering the country’s limited progress in implementing food marketing regulations, these findings highlight the need to develop effective policies aimed at reducing the prevalence of such advertisements. These measures should form part of a broader, comprehensive strategy to discourage the consumption of ultra-processed products.

## Supporting information

Ares et al. supplementary material Ares et al. supplementary material

## Supplementary material

For supplementary material accompanying this paper visit https://doi.org/10.1017/S1368980025000254
