# Supplementary material for: Advertisements of ultra-processed products outside food outlets: field evidence from Montevideo, Uruguay
Source: Public Health Nutr. 2025 Mar 19;28(1):e60. doi: 10.1017/S1368980025000254 (PMC12086735; doi:10.1017/S1368980025000254)
Supplement: Ares et al. supplementary material [file S1368980025000254sup001.docx]

**Supplementary material**

**Table 1.** List of categories frequently regarded as ultra-processed products^(33)^ provided to observers during training.

| **Category** |
| --- |
| Alfajor |
| Breakfast cereals |
| Burgers and other reconstituted meat products |
| Candies, chewing gum and any other type of confectionery |
| Cereal bars |
| Chocolate |
| Cold cuts, hot dogs and sausages |
| Cookies |
| Dressings (e.g., mayonnaise, ketchup) |
| Energy drinks |
| Flavoured milk |
| Flavoured water |
| Frozen pre-fried potatoes |
| Ice-cream |
| Margarine |
| Mass-produced packaged breads and buns |
| Milk desserts |
| Noodles or fresh packaged pasta |
| Packaged fruit juices |
| Packaged pastries, cakes, and powdered cake mixes |
| Poultry and fish nuggets and sticks |
| Powdered and packaged ‘instant’ soups |
| Powdered drinks |
| Pre-prepared ready-to-heat products (e.g., pies, pasta and pizza dishes) |
| Soft drinks |
| Sweet or savoury packaged snacks |
| Yogurt |
